# ErbB2 signaling at the crossing between heart failure and cancer

**DOI:** 10.1007/s00395-016-0576-z

**Published:** 2016-09-05

**Authors:** Zarha Vermeulen, Vincent F. M. Segers, Gilles W. De Keulenaer

**Affiliations:** 1 Laboratory of Physiopharmacology, University of Antwerp, Universiteitsplein 1, 2610 Antwerp, Belgium; 2 Department of Cardiology, University Hospital Antwerp, Wilrijkstraat 10, 2650 Edegem, Belgium; 3 Department of Cardiology, Middelheim Hospital, Lindendreef 1, 2020 Antwerp, Belgium

**Keywords:** Neuregulin-1, ErbB2 signaling, Cardiotoxicity

## Abstract

The dual role of ErbB2 (or HER-2) in tumor growth and in physiological adaptive reactions of the heart positions ErbB2 at the intersection between cancer and chronic heart failure. Accordingly, ErbB2-targeted inhibitory therapy of cancer may lead to ventricular dysfunction, and activation of ErbB2 for heart failure therapy may induce malignancy. The molecular processes leading to the activation of ErbB2 in tumors and cardiac cells are, however, fundamentally different from each other. Thus, it must be feasible to design drugs that specifically target either physiological or malignant ErbB2 signaling, to activate ErbB2 signaling in heart failure with no increased risk for cancer, and to inhibit ErbB2 signaling in cancer with no increased risk for heart failure. In this review, we present a state-of-the-art on how ErbB2 is regulated in physiological conditions and in tumor cells and how this knowledge translates into smart drug design. This leads to a new generation of drugs interfering with ErbB2 in a unique way tailored for a specific clinical goal. These exciting developments at the crossing between cancer and heart failure are an elegant example of interdisciplinary collaborations between clinicians, physiologists, pharmacologists, and molecular biologists.

## Introduction

**Fig. 1:**
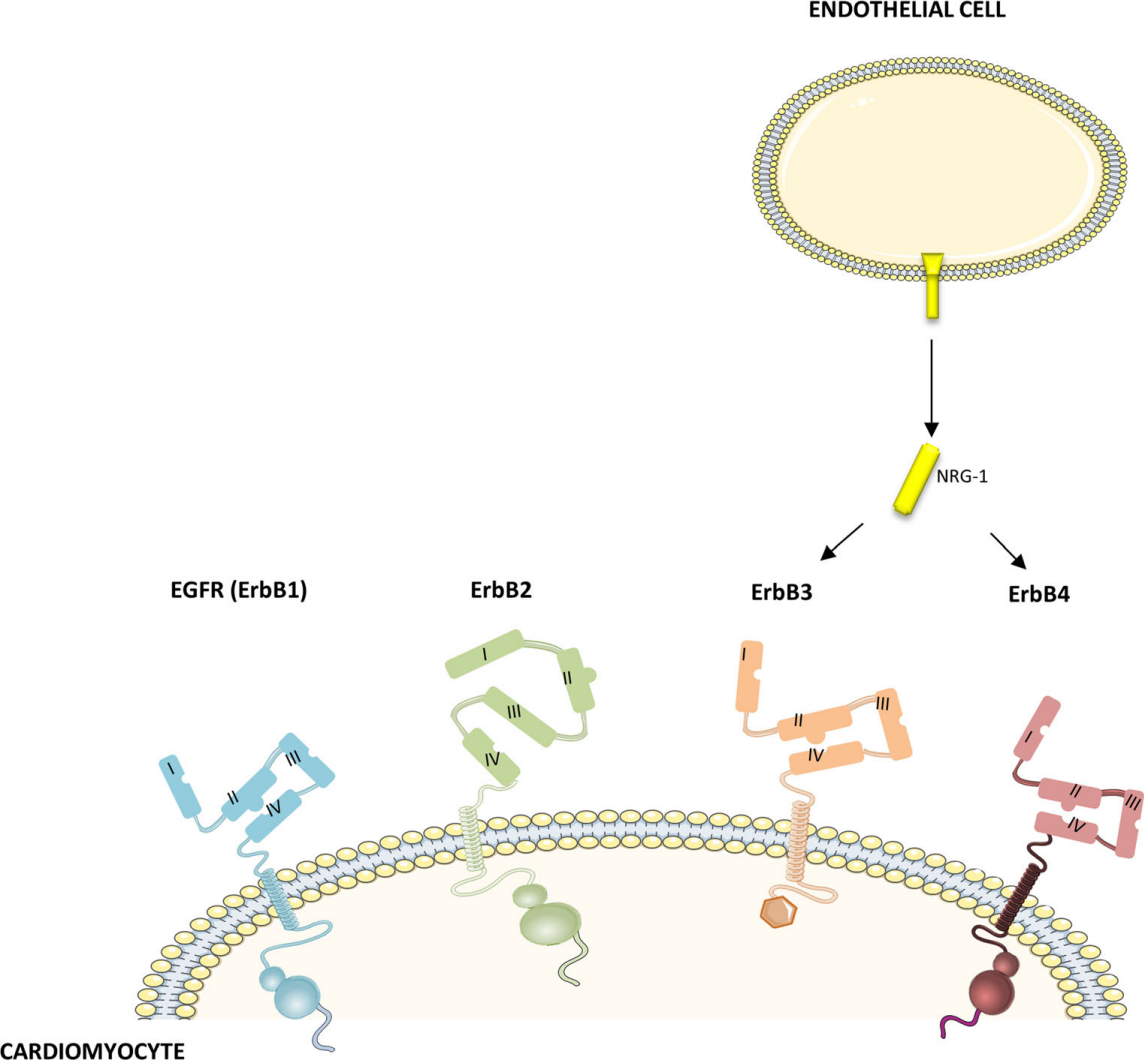
ErbB receptors. Human epidermal growth factor receptors consist of EGFR (ErbB1), ErbB2, ErbB3, and ErbB4. ErbB receptors share high homology in the extracellular domain and the kinase domain. However, ErbB3 lacks tyrosine kinase activity and ErbB2 has no known ligand, instead, it has a constitutive open conformation, continuously exposing a dimerization arm for interaction with another ErbB family member. NRG-1, secreted from cardiac endothelial cells, can bind to ErbB3 and ErbB4

The ErbB2 (or HER2) receptor is overexpressed in 25 % of breast tumors and is a major drug target in cancer therapy. Trastuzumab (Herceptin^®^) was the first anti-ErbB2 drug to be approved by the FDA. Unexpectedly, patients receiving trastuzumab were found to have a high incidence of heart failure, especially when combined with anthracyclines. This observation led to the discovery of the protective ErbB/NRG-1 system in cardiovascular physiology. The combined role of ErbB2 in malignant tumor growth, and in compensatory processes in the heart, positions ErbB2 at the crossing between cancer and heart failure. Hence, pharmacological inhibition of ErbB2 to treat cancer may induce heart failure, while systemic activation of ErbB2 to treat heart failure may induce malignancy. ErbB2 belongs to the family of human epidermal growth factor (EGF) receptors consisting of EGFR (ErbB1), ErbB2, ErbB3, and ErbB4 (Fig. 1). As opposed to its family members, ErbB2 has no known ligand, instead, it has a permanent open confirmation, continuously exposing a dimerization arm for interaction with another ErbB family member (heterotypic ErbB signaling). ErbB3 is a kinase-impaired receptor and requires another dimerization partner for activation.

Despite this general theme, the principles of ErbB2 signaling in physiological conditions, including the regulation of cardiac function, are fundamentally different from those in cancer cells [10]. This suggests that it must be feasible to specifically interfere with either physiological or oncogenic ErbB2 signaling, to activate ErbB2 signaling in heart failure with no increased risk for cancer on the one hand, and to inhibit ErbB2 signaling in cancer with no increased risk for heart failure on the other hand. Smart drug design developed in a stepwise fashion has now provided at least seven different anti-ErbB2 cancer drugs, together with a few molecules to activate physiological ErbB2 signaling for the treatment of heart failure. Each of these drugs interferes with ErbB2 in a unique way. In this review, we present a comprehensive overview of these developments at the crossing between cancer and heart failure. We will address the biology of ErbB signaling in cancer and cardiac cells, the regulatory aspects and clinical benefits of NRG-1/ErbB signaling activation in heart failure, and the working mechanisms of different anti-ErbB2 drugs and their degree of cardiotoxicity.

### ErbB2 signaling in the heart

Myocardial ErbB2 signaling is elementary during fetal development, adaptation, and, perhaps, even regeneration of the heart [9, 31, 41]. In adult life, myocardial ErbB2 is part of an endothelium-controlled NRG-1/ErbB signaling axis, in which NRG-1, secreted from cardiac endothelial cells, binds to ErbB4 and/or ErbB3 in the myocardial tissue [6, 10]. In neonatal hearts, ErbB2 and ErbB4 are highly expressed, but they progressively decrease 1 week after birth. ErbB3 expression is more stable, and may even be higher throughout the postnatal development [6, 9]. Although NRG-1 signaling has generally been attributed to signaling effects mediated by ErbB4, recent data suggest that ErbB3 cannot be ignored [61]. As opposed to ErbB4, however, ErbB3 cannot signal through homodimers and always requires another ErbB dimerization partner to initiate intracellular signaling.

**Fig. 2:**
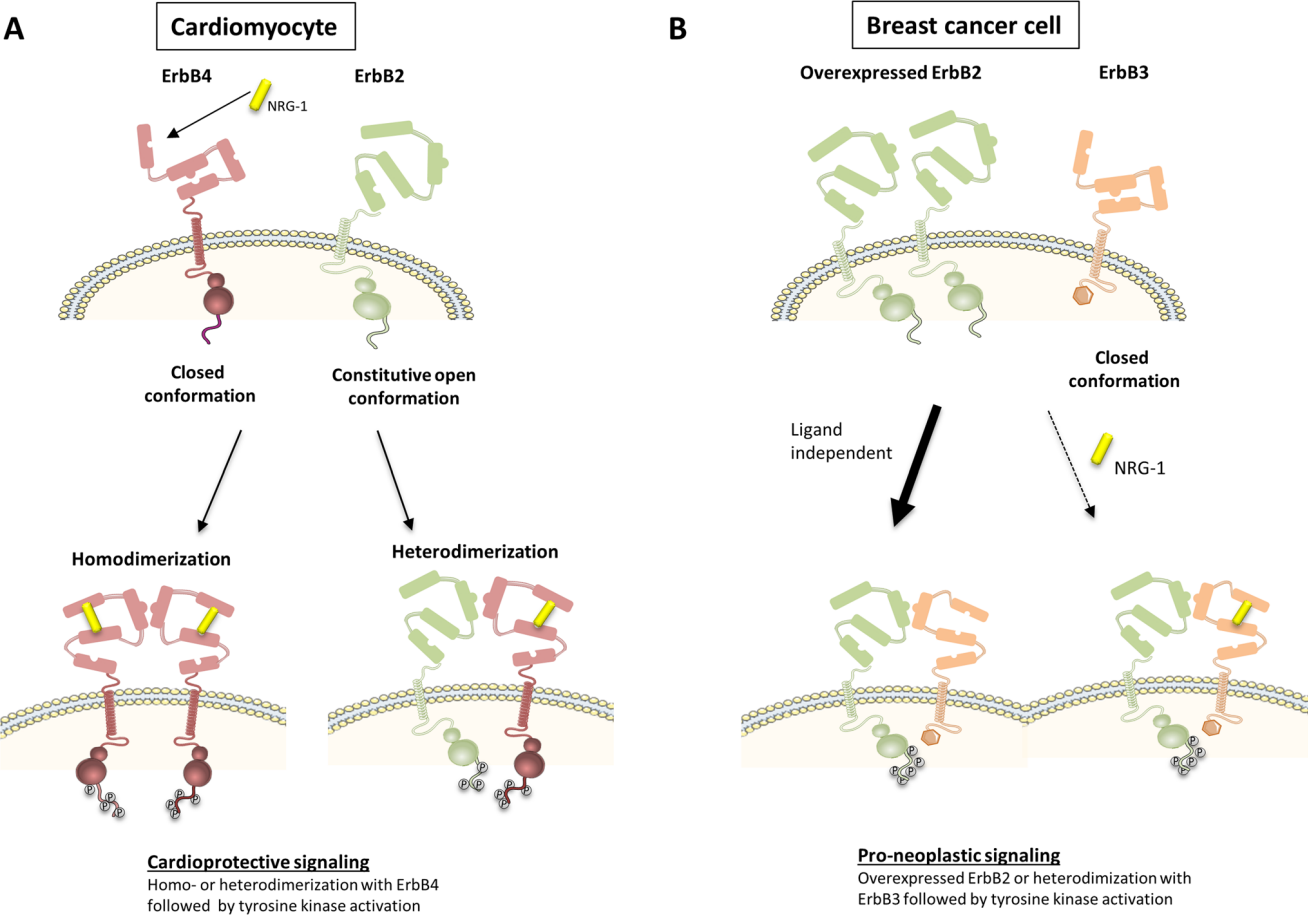
ErbB signaling in ErbB2-overexpressing breast cancer cells and in cardiomyocytes. **a** In adult cardiomyocytes, NRG-1 binding to ErbB4 induces a switch from a closed to the open receptor conformation, exposing a dimerization arm (domain II). Ligand-activated ErbB4 results in the formation of either ErbB2/ErbB4 heterodimers or ErbB4/ErbB4 homodimers. This leads to the phosphorylation of specific tyrosines in the tail region, and the activation of downstream proteins and cardioprotective signaling cascades. **b** Oncogenic signaling in breast cancer cells can be mediated by overexpressed ErbB2. Amplified ErbB2 triggers predominantly ligand-independent oncogenic ErbB2/ErbB3 heterodimers. This leads to the phosphorylation of specific tyrosines in the tail region of the receptors, and the activation of downstream proteins and pro-neoplastic signaling

**Fig. 3:**
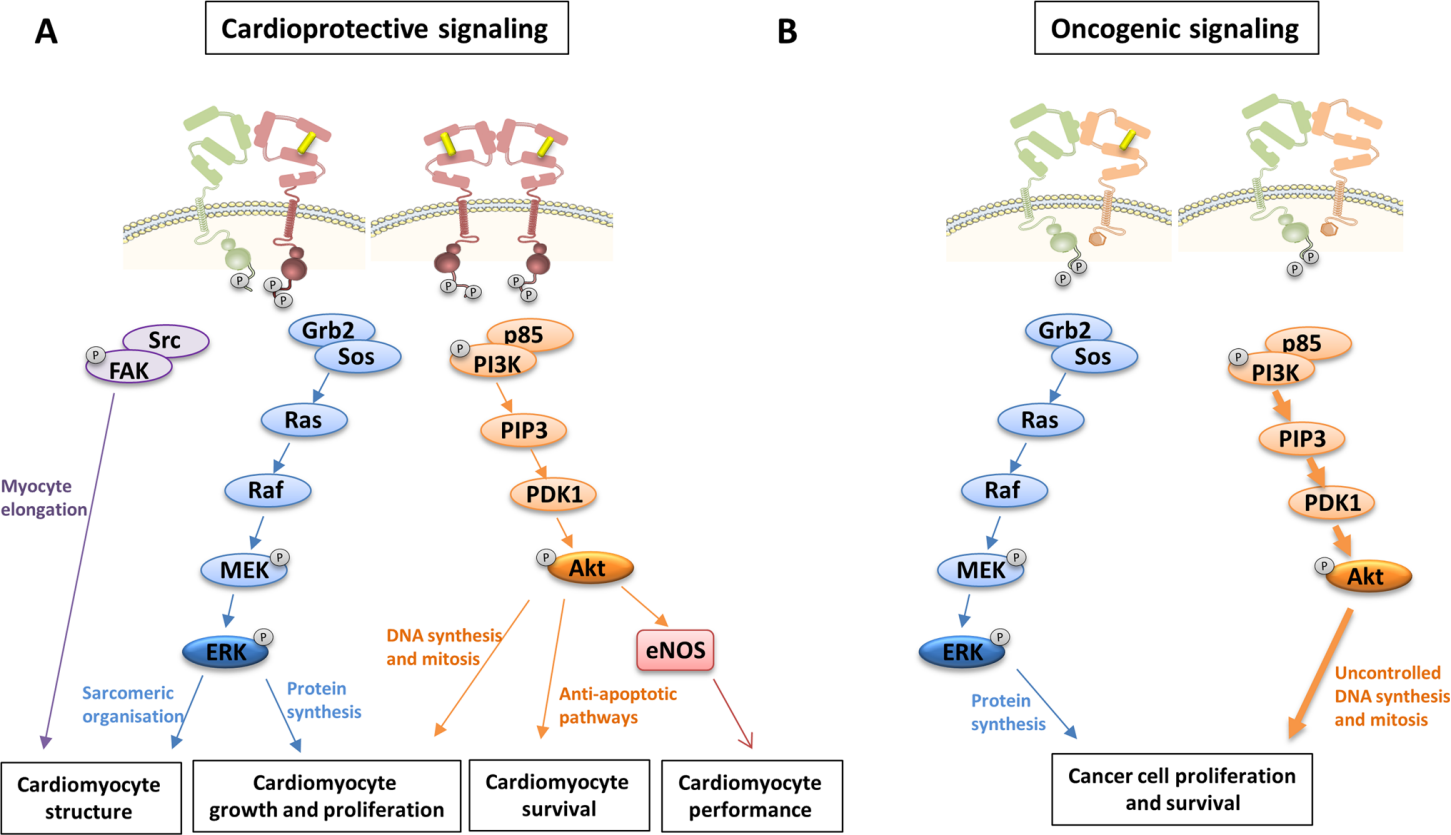
Intracellular signaling cascades following the activation of ErbB receptors in cardiomyocytes and cancer cells. **a** ErbB receptor homo- or heterodimerization in cardiomyocytes triggers the activation of Ras/ERK, PI3K/Akt and Src/FAK signaling pathways. NRG-1/ErbB signaling in cardiomyocytes regulates structure, function, growth, proliferation and survival [1, 39]. **b** Oncogenic signaling through ErbB2 homodimers or ErbB2-ErbB3 heterodimers induces the activation of Ras/ERK and PI3K/Akt signaling pathways. PI3K/Akt signaling has a central oncogenic role as its robust activation induces intense cell growth and proliferation [55]. *Src* proto-oncogene tyrosine-protein kinase, *FAK* focal adhesion kinase, *Grb2* Growth factor receptor-bound protei-2, *Sos* Son of sevenless, *MEK* mitogen-activated ERK kinase, *ERK* extracellular signal-regulated kinase, *PI3K* phosphatidyl inositol-3 kinase, *PIP3* phosphatidyl inositol (4,5,6)-triphosphate, *PDK1* phosphoinositide dependent protein kinase-1, *Akt* protein kinase B, *eNOS* endothelial nitric oxide synthase

Following binding of ErbB4 to NRG-1, ErbB4 undergoes a conformation switch from a closed conformation to an open conformation, exposure of a dimerization arm in subdomain II, formation of ErbB4/ErbB2 heterodimers, increased ErbB4/ErbB2 tyrosine kinase activity, and trans-phosphorylation of the ErbB cytoplasmic signaling tails (Fig. 2a). Although ErbB2 is the preferred dimerization partner of ligand-activated ErbB4, NRG-1 may also induce the formation of ErbB4 homodimers (homotypic ErbB signaling) or ErbB4/ErbB3 heterodimers, and has such signal in an ErbB2-independent way. The relative contribution between both homotypic and heterotypic signaling by NRG-1 is unknown. It is also not known whether the relative contribution of homo- and heterodimer signaling and the ratio between ErbB2, ErbB3, and ErbB4 may change in certain conditions. As further explained below, experiments with bivalent NRG-1 have shown that homotypic NRG-1 signaling (through ErbB4/ErbB4 homodimers) may be sufficient for cardioprotection [27]. Receptor homo- or heterodimerization triggers the activation of signaling pathways, such as the phosphoinositide 3-kinase (PI3K)/Akt, Ras/Extracellular signal-regulated kinases (ERK), and proto-oncogene tyrosine-protein kinase (Src)/focal adhesion kinase (FAK) pathway. NRG-1/ErbB signaling protects cardiomyocytes from apoptosis and stimulates NO production through PI3K/Akt signaling. It also induces cardiomyocyte growth and proliferation through both PI3K/Akt and ERK1/2 signaling (Fig. 3a) [47].

The NRG-1/ErbB signaling axis is activated in heart failure, and compensates for maladaptive processes that lead to the progression of cardiac dysfunction, at least during the early stages of the syndrome [30, 31]. Recently, D’Uva et al. showed that the mechanism of ErbB2-mediated cardioprotection may be subdivided into regeneration by increased cardiomyocyte dedifferentiation and proliferation (which was ERK-dependent), and the induction of hypertrophy and cell survival (which is both Akt- and ERK-dependent) [9]. They also showed that transient reactivation of ErbB2 signaling after myocardial infarction promotes heart regeneration, which after ErbB2 signaling termination, resulted into cardiomyocyte redifferentiation and tissue replacement with reduced scarring [9]. This study suggests that tuning ErbB signaling in the correct way could provide an optimal route for maximizing endogenous regeneration capacity of the injured adult heart. Of note, Kühn et al. demonstrated that these regenerative effects of NRG-1 are more pronounced during the neonatal period than in adulthood [44]. Interestingly, another series of recent experiments indicate that, apart from healing the heart, the activation of ErbB signaling with systemic NRG-1 may also mitigate common co-morbidities of heart failure, including diabetes, atherosclerosis, pulmonary hypertension, and renal dysfunction [12, 40, 56, 59]. These effects are most likely mediated by acting on local tissue ErbB receptors in the liver, skeletal muscle cells, kidney cells, and vascular cells [42, 56]. Accordingly, apart from its effects on the failing heart, NRG-1 may have pleiotropic actions in several organs and pathophysiological processes throughout the organism. If confirmed in humans, on top of the standard therapy, NRG-1 treatment may take a particular position in the pharmacological therapy of heart failure aimed at regenerating the injured heart and mitigating its co-morbidities. NRG-1 has been used in many animal models of heart failure and is currently tested in phase III clinical trial in heart failure with a reduced left ventricular ejection fraction (Table 1-2) [41]. In these trials, NRG-1 is administrated as EGF-domain fragment of recombinant human (rh)NRG-1β or as an Ig domain-containing version of NRG-1β known as glial growth factor 2 (GGF2), usually by intravenous infusions.

**Table 1:** Administration of rhNRG-1 in animal models of heart failure

Model	Species		RhNRG-1 treatment	Outcome
MI [37]	Rat		10 μg/kg day i.v. for 5 or 10 days, 1 week or 2 months after LAD ligation	Improved LV structure and dysfunction Increased angiogenesis Improved survival
MI [2]	Mouse		2.5 μg/mouse i.p. for 12 weeks, 1 week after LAD	Improved LV structure and dysfunction Increased myocardial regeneration
MI [19]	Rat		5 μg/kg h i.v. for 7 days, 8 weeks after LAD ligation	Improved LV structure and dysfunction
MI [20]	Rat		10 μg/kg day i.v. for 10 days, 4 weeks after LAD ligation	Improved LV structure and dysfunction Decreased mitochondrial dysfunction Decreased apoptosis Decreased oxidative stress
MI [58]	Rat		Infarcted area injected with rhNRG-1 carrying lentivirus	Decreased apoptosis Increased angiogenesis
MI [15]	Swine		0.67 mg/kg i.v. every 2 days for 4 weeks, 1 week after MI	Improved LV dysfunction Decreased fibrosis
I/R [13]	Rat		1, 2, 4 or 8 μg/kg i.v. for 20 min prior to I/R	Improved LV structure Decreased apoptosis Decreased infarct size
Myo-carditis [37]	Mouse		30 μg/kg day i.v. for 5 days	Improved LV structure and dysfunction Decreased necrosis Improved survival
Doxo-induced CM [37]	Rat		20 μg/kg.day i.v. for 5 days, 4 weeks after first doxorubicin administration	Improved LV structure and dysfunction Decreased necrosis Improved survival
Doxo-induced CM [3]	Mouse		0.75 mg/kg day s.c. for 3–5 days, 1 day before doxo administration	Improved LV structure and dysfunction Improved survival Decreased apoptosis Preserved cardiac troponins
Pacing-induced CM [37]	Dog		3 μg/kg day i.v. for 5 days with continuous pacing, 3 weeks after starting rapid pacing	Improved LV dysfunction
Pacing-induced CM [34]	Rhesus monkey		3 µg/kg day i.v. for 10 days	Improved LV dysfunction Increased myosin heavy chain α
Type 1 DCM [33]	Mouse		10 μg/kg i.v. every 2 days for 2 weeks, 12 weeks after STZ injection	Improved LV structure and dysfunction Decreased apoptosis Decreased fibrosis

*RhNRG-1* Recombinant human neuregulin-1, * MI* Myocardial infarction, * I.v.* Intravenous,* LAD* Left anterior descending artery,* LV* Left ventricle,* I.p.*, Intraperitonea,* I/R* Ischemia/Reperfusion,* Doxo* Doxorubicin,* S.c.* Subcutaneous, *CM* Cardiomyopathy,* DCM* Diabetic cardiomyopathy,* STZ* Streptozotocin

**Table 2:** Clinical trials with rhNRG-1 as a therapy for heart failure

Description	Dosage	Outcome
Phase II, randomized, double-blind, multicenter, background-therapy-based, placebo-controlled, parallel group study [17]	0.3, 0.6 or 1.2 μg/kg 10 h i.v. infusion, 10 consecutive days	Improved and sustained LVEF % and decreased LVEDV and LVESV at 30 and 90 days after treatment
Single-center, prospective, non-randomized, open label study [25]	Initial dose of 1.2 μg/kg for 6 h 0.6, 1.2 or 2.4 μg/kg for 12 h i.v. infusion, 10 consecutive days	Acute increase in CO Improvement in LVEF

*I.v.* Intravenous, * LVEF %* % Left ventricle ejection fraction, * LVEDV* Left ventricle end-diastolic volume, * LVESV* Left ventricle end-systolic volum, * CO* Cardiac output

A concern during pharmacological ErbB signaling activation in heart failure is, however, a potential stimulation of tumor growth. As explained below, tumor growth in ErbB2-amplified cells mainly results from the ligand-independent formation of ErbB2/ErbB3 oncogenic complexes. Therefore, at first glance, NRG-1 should not induce oncogenic complexes, and the risk to induce malignancy should be limited. However, NRG-1 also binds to ErbB3 on cancer cells forcing it to the “open conformation” and favoring formation of new oncogenic complexes. In this scenario, NRG-1 may advance tumor growth and/or promote tumor resistance during treatment with anti-ErbB2 therapies. A recent study contradicts this hypothesis. Ganapathy et al. demonstrated that rhNRG-1 administration in the first month of life, sufficient for stimulating cardiac regeneration, did not induce unwanted extra-cardiac neoplastic growth or the early stage neoplastic foci within 6 months [16].

### Smart drug design to activate ErbB signaling without inducing cancer

**Fig. 4:**
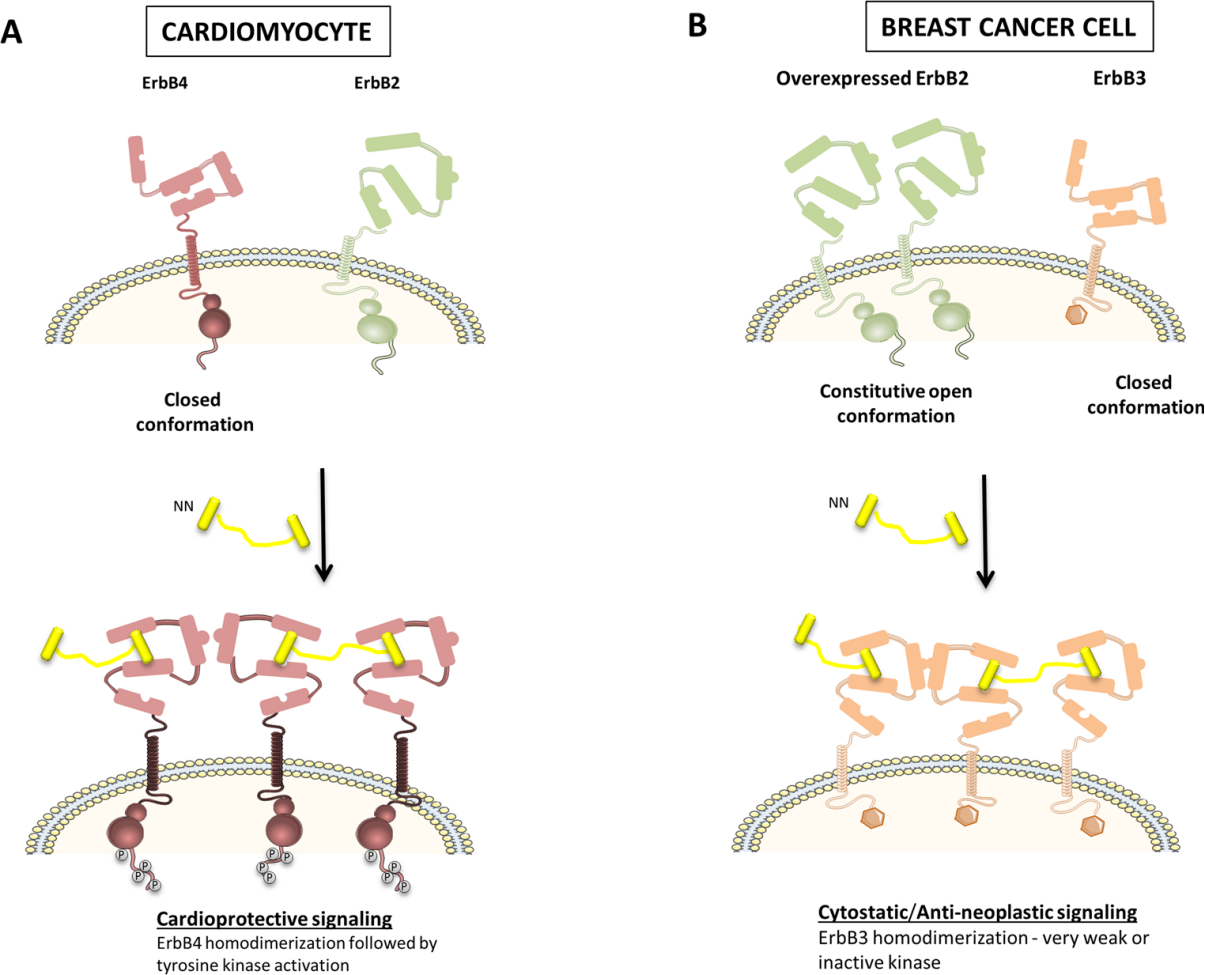
Effect of bivalent NRG-1β (ΝΝ) in ErbB2-overexpressing breast cancer cells and cardiomyocytes. **a** In cardiac cells, NN will predominantly promote homotypic association of ErbB4, but signaling through ErbB2/ErbB4 heterodimers will be reduced. ErbB4 homotypic signaling seems to be sufficient for cardioprotective signaling. **b** In tumor cells, ΝΝ drives a stable, homotypic association of ErbB3, which traps ErbB3, keeping it away from undesirable oncogenic signaling with ErbB2. ErbB3 has a weak kinase activity, and hence, ErbB3 homodimers are incapable of activating downstream signaling pathways. Consistently, NN induces anti-neoplastic or cytostatic responses in cancer cells

In an attempt to design a translationally relevant ErbB agonist for the treatment of chronic diseases, such as heart failure, Griffith and Lee created bivalent NRG-1β (ΝΝ) [26, 27]. NN is a ligand for both ErbB3 and ErbB4 and drives particular homotypic interactions at the expense of others. This alters signaling and phenotypic outcomes compared with their native, monovalent counterparts. In cardiac cells, NN will predominantly promote the homotypic association of ErbB4, thereby reducing signaling through ErbB2/ErbB4 heterodimers (Fig. 4a). In tumor cells, NN drives a stable, homotypic association of ErbB3, which traps ErbB3, keeping it away from undesirable oncogenic signaling with ErbB2. ErbB3 has a weak kinase activity, and hence, ErbB3 homodimers are incapable of activating downstream signaling pathways (Fig. 4b). Consistently, NN induced anti-neoplastic or cytostatic responses in cancer cells [27]. The differences between the action of monovalent NRG-1 and bivalent NN are summarized in Table 3. Subsequent studies showed that NN significantly attenuated doxorubicin-induced cardiac dysfunction, indicating that ErbB4 homodimer signaling suffices for cardioprotection or, alternatively, that there remains enough ErbB2/ErbB4 signaling for a normal activity [27].

**Table 3:** Differences in the mechanism of monovalent NRG-1 compared with bivalent NRG-1

Drug	Tumor cell	Cardiomyocyte
Neuregulin-1 (NRG-1)	NRG-1 stimulation may increase malignant potential of ErbB2-expressing cancer cells by inducing ErbB2 and ErbB3 heterotypic, pro-neoplastic interactions	NRG-1 is cardioprotective by inducing ErbB2/Erbb4 heterotypic and ErbB4/ErbB4 homotypic interactions
Bivalent NRG-1 (NN)	NN decreases migration, inhibits proliferation and increases apoptosis in ErbB2-expressing cancer cells by trapping ErbB3 for homotypic, weak/non-signaling interactions	NN is cardioprotective by inducing ErbB4 homotypic interactions

Accordingly, given the reduced pro-neoplastic potential of NN versus rhNRG-1, NN has translational potential for the treatment of chronic diseases, such as heart failure, with no increased risk to induce cancer.

### ErbB2 signaling in cancer

In many different cancer cells, ErbB2 is amplified by increased gene transcription. Tumor growth is critically dependent on ErbB2 amplification, a process coined oncogenic addiction of malignancy. Amplified ErbB2 can bind to ErbB3 in an uncontrolled and at least partially ligand-independent way, forming an oncogenic ErbB2/ErbB3 complex (Fig. 2b) [22]. In this complex, following phosphorylation of ErbB3 tyrosine residues by the ErbB2 kinase, ErbB3 interacts with the regulatory p85 subunit of PI3K without requirement of any adaptor proteins [46]. This results in the robust activation of the PI3K/Akt pathway and intense cell growth and proliferation, positioning ErbB3 as the key node in oncogenic ErbB2 signaling (Fig. 3b). This scenario is identified in 20–30 % of invasive breast carcinomas, and in significant cases of ovarian, gastric, and bladder cancer.

ErbB2 has been the main therapeutic target within the ErbB2/ErbB3 oncogenic unit. The goal of ErbB2-targeted therapy is to interrupt PI3K/Akt signaling to stop cell proliferation and induce cell apoptosis. Clinical introduction of this strategy with the humanized anti-ErbB2 antibody trastuzumab (Herceptin^®^) has been successful, but chronic treatment has been disturbed by unforeseen problems, namely, (a) the development of tumor drug resistance and (b) the induction of cardiac dysfunction and heart failure [23]. These drawbacks have forced the design of newer anti-ErbB2 drugs.

**Fig. 5:**
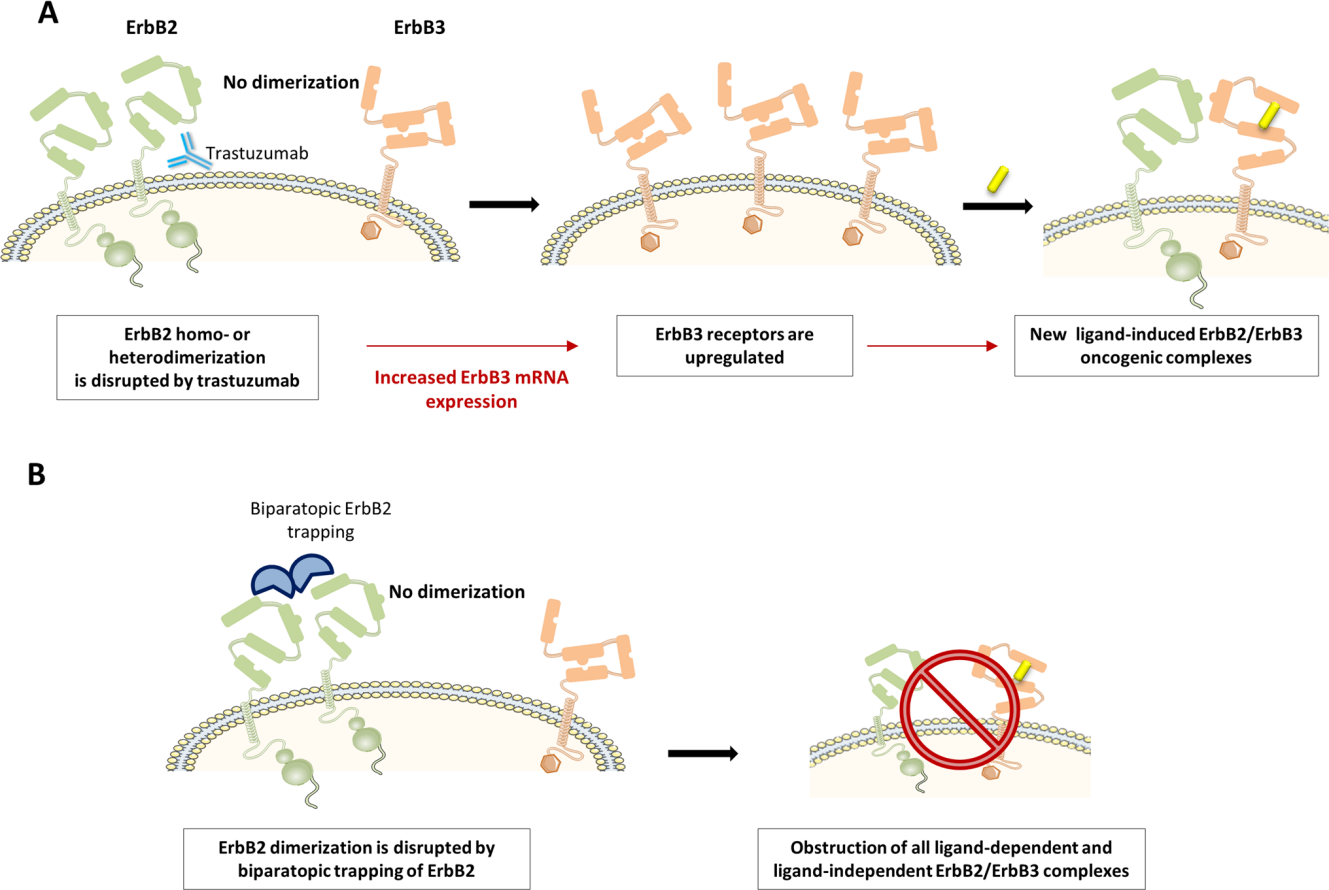
Trastuzumab resistance. **a** Trastuzumab provides an ErbB3 escape route for the development of tumor resistance. When inhibiting ErbB2 signaling, ErbB3 expression will be upregulated to form new oncogenic complexes with ErbB2, especially when NRG-1 or another ErbB3 ligand is available to open its conformation. Consistently, both the upregulation of ErbB3 mRNA and the concentration of NRG-1 in tumor predict trastuzumab drug resistance. **b** Biparatopic ErbB2 trapping blocks all ligand-dependent and ligand-independent dimers of ErbB2. Pan-ErbB2 inhibition will stop PI3K/Akt signaling and avoid compensatory responses that lead to drug resistance

A first challenge has been to intercept biological escape routes leading to anti-ErbB2 drug resistance. Recent evidence suggests that the upregulation of ErbB3 is critically involved in the process [51]. ErbB3 mRNA transcription is negatively controlled by ErbB2 signaling, explaining why ErbB3 is upregulated during the suppression of ErbB2 signaling (Fig. 5a) [18]. Enhanced ErbB3 forms new oncogenic complexes with residually active ErbB2, which maintains oncogenic signaling and endorses anti-ErbB2 drug resistance [46]. This mechanism is especially intense when autocrine or paracrine NRG-1 is available in the tumor [45]. When NRG-1 binds to ErbB3, its conformation switches to the open conformation as such boosting dimerization of ErbB3 with the remaining active ErbB2. Thus, to avoid drug resistance, it seems compulsory to combine specific anti-ErbB2 therapy with anti-ErbB3 interventions, or to engineer bispecific anti-ErbB drugs that inhibit both ErbB2 and ErbB3 in a combined fashion. A recent study showed that trapping ErbB2 in a dimerization-incompetent state will also result into obstruction of all ligand-dependent and ligand-independent ErbB2/ErbB3 complexes (Fig. 5b). This pan-ErbB2 inhibition was shown to disarm all compensatory mechanisms and, therefore, to avoid the development of drug resistance [55].

A second challenge has been to avoid anti-ErbB2 cardiotoxicity. As explained above, ErbB2 signaling is important for cardiac physiology, especially in the conditions of cardiac overload and injury. However, as opposed to tumor cells, cardiac ErbB2 signaling is critically ligand (NRG-1)-dependent. This suggests that, as long as inhibitory anti-ErbB2 treatment does not interfere with ligand-dependent ErbB2 signaling, its cardiac profile may be safe [10].

### Trastuzumab: The first ErbB2-targeted drug, how does it work, where does it fail?

#### The clinical picture

Trastuzumab (Herceptin^®^) is an effective anticancer treatment for ErbB2-overexpressing breast cancer in the adjuvant, neo-adjuvant, and metastatic settings. Adding the conventional chemotherapeutics increases the overall response to trastuzumab. In the adjuvant setting, based on several phase III trials, treatment with trastuzumab is given for 1 year. In the first human trials of trastuzumab, however, an unexpected cardiac toxicity was identified: 27 % of patients receiving trastuzumab concurrently with anthracycline-containing chemotherapy developed either asymptomatic cardiomyopathy or clinical heart failure compared with 7 % of patients receiving anthracycline chemotherapy alone [17, 49, 52]. Fortunately, in many cases, the cardiac dysfunction was asymptomatic, and further clinical trials indicated that in the absence of concomitant anthracycline treatment, the incidence of cardiac dysfunction was relatively low during treatment with trastuzumab, generally transient after the completion of therapy. In addition, the cardiac risks were outweighed overall by the potent anticancer effect of trastuzumab treatment [1].

**Fig. 6:**
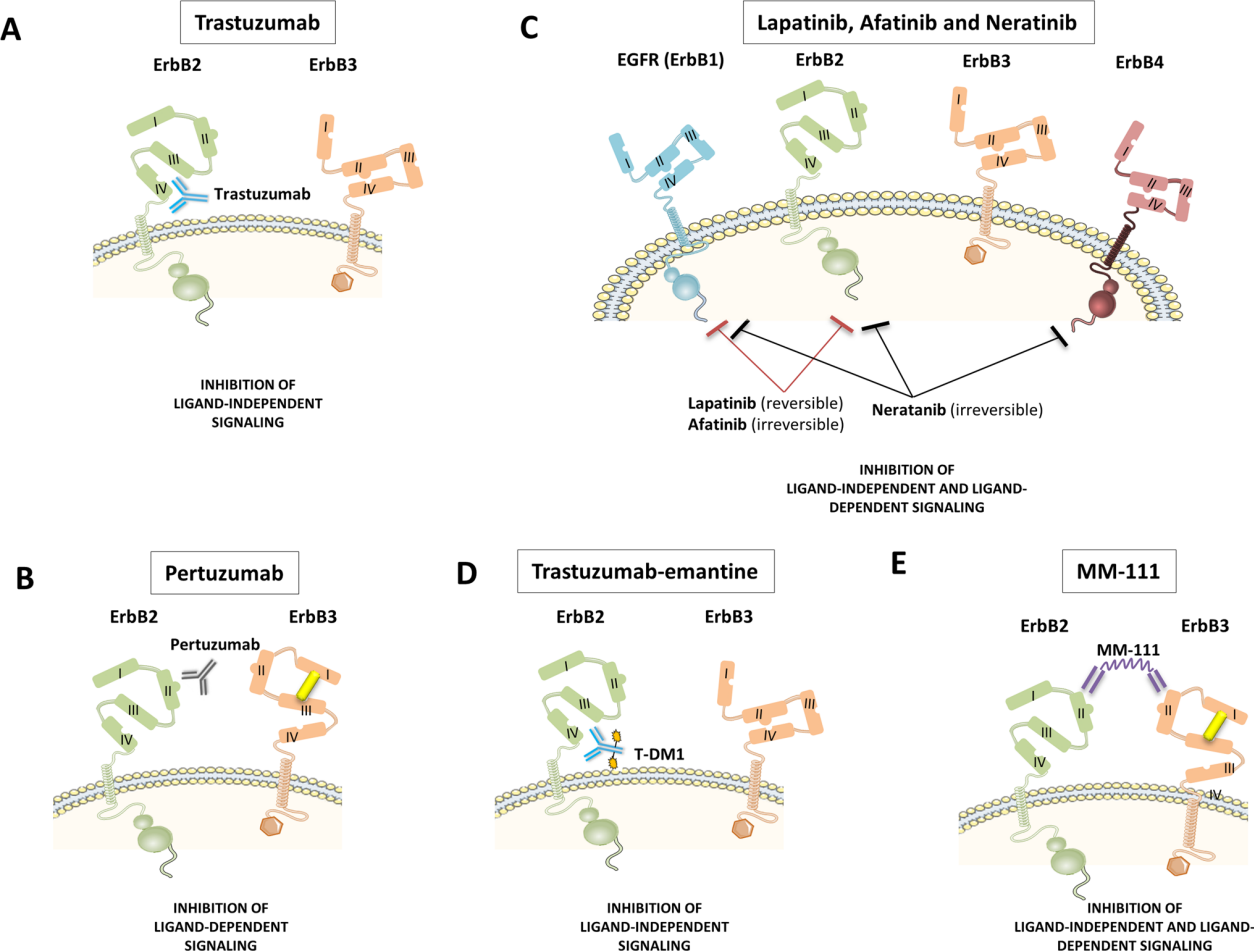
Primary actions of current ErbB2 inhibitors. **a** Trastuzumab is a humanized monoclonal antibody to subdomain IV of ErBb2. This leads to the inhibition of ligand-independent ErbB2 signaling. **b**. Pertuzumab is a humanized monoclonal antibody to subdomain II of the dimerization arm of ErbB2. Pertuzumab leads to the inhibition of ligand-induced ErbB2 signaling. **c**. Lapatinib is a small molecule tyrosine kinase inhibitor of EGFR (ErbB1) and ErbB2. Afatinib and neratinib are two second-generation irreversible tyrosine kinase inhibitors. Afatinib is highly selective for EGFR/ErbB1 and ErbB2, whereas neratinib also inhibits ErbB4. Tyrosine kinase activity is blocked independently of whether this activity is ligand-induced or not. **d** Trastuzumab-emtansine is an antibody conjugate consisting of the monoclonal antibody trastuzumab linked to cyctotoxic agent emtansine, an antimicrotubule drug. **e** MM-111 is a bispecific antibody and polypeptide fusion protein of two human scFv antibodies linked to modified human serum albumin. MM-111 forms a trimeric complex with ErbB2 and ErbB3, inhibiting ligand-dependent ErbB2 and ErbB3 signaling

Trastuzumab is a monoclonal antibody that binds to subdomain IV of ErbB2 (Fig. 6a). This binding disrupts ligand-independent ErbB2/ErbB3 interactions in ErbB2-amplified cells and subsequently impedes PI3K/Akt activity [23, 28]. However, it is yet uncertain whether this is the only mechanism responsible for trastuzumab’s in vivo cancer-inhibiting actions. Other proposed mechanisms include increased endocytotic destruction of ErbB2, reduced shedding of the extracellular domain of ErB2 and immune activation by recruiting Fc-competent immune effector cells, and other components of antibody-dependent cell-mediated cytotoxicity (ADCC) [23].

Due to the monoclonal humanized nature of trastuzumab, this antibody can only be studied on human cells. This explains why it has remained long unclear whether trastuzumab also interferes with NRG-1-dependent physiological ErbB signaling in the heart. Recently, however, using human fetal cardiomyocytes, Fedele and coworkers showed that trastuzumab, indeed, inhibited the formation of NRG-1-induced ErbB4/ErbB2 complexes [14]. This observation most likely explains why trastuzumab’s cardiac toxicity primarily occurs when it is given simultaneously with anthracyclins. Indeed, NRG-1/ErbB signaling in the heart is part of a stress-activated compensatory system, playing a modest role in physiological conditions, but becoming indispensable in the injured heart, e.g., during ischemia or exposure to cardiotoxic agents, such as anthracyclins [21, 36].

Another disadvantage of trastuzumab’s working mechanism is that it provides an ErbB3 escape route for the development of tumor resistance. Upon inhibition of ErbB2 signaling, ErbB3 expression is upregulated, and forms new oncogenic complexes with ErbB2, especially when NRG-1 or another ErbB3 ligand is available to open its conformation. Consistently, both the upregulation of ErbB3 mRNA and the concentration of NRG-1 in tumor predict trastuzumab drug resistance (Fig. 5) [46].

Accordingly, trastuzumab is an effective drug to inhibit malignant signaling in ErbB2 overexpressing tumor cells, but it has the disadvantage of (a) interfering with NRG-1/ErbB2 signaling in the heart, which becomes harmful during co-treatment with antracyclines, and (b) allowing an ErbB3-dependent escape route for drug resistance through the upregulation of ErbB3 and formation of ligand-induced ErbB2/ErbB3 oncogenic complexes.

### How do next generation ErbB2 antagonists differ from trastuzumab?

During the past decade, we have witnessed the introduction of several new drugs to target ErbB2 in cancer. These drugs include new monoclonal antibodies against ErbB2 (pertuzumab, MM-111), oral small molecules ErbB tyrosine kinase inhibitors (lapatinib, afatinib, and neratinib), and an antibody–drug conjugate of trastuzumab combined with a cytotoxic agent, emtansine. Each of these molecules has a specific mode of action at the level of the ErbB receptors, hence influencing ligand-dependent and ligand-independent ErbB signaling in a unique way (Fig. 6). Specific activity profiles allow predicting their effect on tumor growth, the potential development of drug resistance, and their interference with physiological NRG-1-induced ErbB signaling in the heart and other organs. In the next paragraphs, we have compiled these profiles, and summarized them in Table 4.

**Table 4:** Action of different ErbB2 antagonist on cancer cells and on ligand (NRG-1)-induced signaling and on cardiac function

Drug	Tumor cell		Cardiomyocyte	
	Anticancer mechanism	Secondary Resistance	Effect on NRG-1 signaling	Cardiotoxicity
Trastuzumab	Inhibition of ErbB2/ErbB3 dimerization by binding domain IV of the ErbB2 receptor	Yes	Inhibits heterotypic ErbB2/ErbB4 signaling, but not homotypic ErbB4/ErbB4 signaling	Tastuzumab monotherapy: [49, 53] 7 % Trastuzumab with anthracyclines: [52] 27 %
Pertuzumab	Inhibition of ErBb2/ErbB3 dimerization by binding domain II of the ErbB2 receptor	No	Inhibits heterotypic ErbB2/ErbB4 signaling, but not homotypic ErbB4/ErbB4 signaling	Pertuzumab monotherapy: [32] <7 %
Lapatinib	Reversible tyrosine kinase inhibitor of EGFR/ErbB1 and ErbB2	Yes [8]	Inhibits heterotypic ErbB2/ErbB4 signaling, but not homotypic ErbB4/ErbB4 signaling	Lapatinib monotherapy: [43] <2 %
Neratinib	Irreversible tyrosine kinase inhibitor of EGFR/ErbB1, ErbB2 and ErbB4	Unknown	Inhibits heterotypic ErbB2/ErbB4 signaling and potentially homotypic ErbB4/ErbB4 signaling	None reported [4, 48]
Afatinib	Irreversible tyrosine kinase inhibitor of EGFR/ErbB1 and ErbB2	Unknown	Inhibits heterotypic ErbB2/ErbB4 signaling, but not homotypic ErbB4/ErbB4	None reported [35, 60]
Trastuzumab-emtansine (T-DM1)	Trastuzumab activity combined with intracellular delivery of a microtubule depolymerisation agent (emtansine)	Unknown	Inhibits heterotypic ErbB2/ErbB4 signaling, but not homotypic ErbB4/ErbB4 signaling	T-DM1 monotherapy: [57] <2 %
MM-111	A bispecific molecule targeting the ErbB2/ErbB3 heterodimer, forming an inactive complex	Unknown	No binding in the heart given absence of ErbB2/ErbB3 heterodimers	None

### Pertuzumab

#### The clinical picture

The combination of pertuzumab plus trastuzumab plus docetaxel, as compared with placebo plus trastuzumab plus docetaxel, when used as the first-line treatment for ErbB2-positive metastatic breast cancer, significantly prolongs progression-free survival. In the phase III randomized, double-blind, multinational CLEOPATRA trial, pertuzumab plus trastuzumab did not increase cardiac toxic effects [54]. Pertuzumab has not been tested in co-administration with anthracyclins.

Pertuzumab is a monoclonal antibody binding to subdomain II of ErbB2, hence, interfering specifically with ligand-induced ErbB2 dimerization (Fig. 6b) [10]. This working mechanism makes it a perfect drug to be combined with trastuzumab, as it will complementary inhibit the formation of ligand-induced ErbB2/ErbB3 oncogenic complexes arising after the upregulation of ErbB3 by trastuzumab [28]. This phenomenon most likely explains the success of the combination of trastuzumab and pertuzmab in the clinic: a prolonged survival of patients with metastatic breast cancer when pertuzumab was added to a treatment with trastuzumab and anthracyclines [7].

In contrast to trastuzumab, pertuzumab will also interfere with physiological ligand-induced ErbB2 signaling and as such potentially abrogate the protective actions of NRG-1 in the heart [28]. In fact, ErbB2 signaling in non-amplified ErbB2 cells may be abrogated more efficiently than in tumor cells, given the lower amount of ErbB2 copies. However, pertuzumab does not impede ligand-induced signaling through ErbB4 homodimers or through ErbB4/ErbB3 heterodimers. It seems plausible that in the presence of pertuzumab and a reduced availability of ErbB2′s dimerization arms, physiological NRG-1 signaling shifts towards ErbB2-independent signaling. Pertuzumab may thus merely modify but not inhibit physiological ErbB signaling activity. These phenomena may explain why in clinical trials, pertuzumab has emerged as a safe drug, with little or no cardiac toxicity.

### Lapatinib

#### The clinical picture

Lapatinib is generally reserved for the late-stage treatment, and only in combination with capecitabine for ErbB2-positive breast cancer in women, whose cancer has progressed following previous chemotherapy with anthracycline, taxanes, and trastuzumab. In combination with capecitabine, reversible decreases of left ventricular function occur [5].

Lapatinib is a small molecule intracellular and reversible ErbB2- and EGFR/ErbB1 tyrosine kinase inhibitor (Fig. 6c) [10]. As such, compared with trastuzumab, lapatinib has the advantage of inhibiting the phosphorylation of ErbB3 by ErbB2 in the oncogenic complex, independently of whether this complex was formed in a ligand-dependent or independent way. Hence, single treatment with lapatinib leaves little room for the development of drug resistance through ligand-induced ErbB2/ErbB3 oncogenic complexes, thereby mimicking the combination of trastuzumab plus pertuzumab. Nevertheless, recent studies indicate that ErbB3 is upregulated in lapatinib-treated cells and that it may still form ErbB2/ErbB3 complexes with residually active ErbB2 [38].

With regard to its effect on physiological NRG-1/ErbB signaling, lapatinib clearly abrogates ligand-induced ErbB2 signaling, hence potentially interfering with NRG-1-induced signaling. Similarly, as for pertuzumab, however, lapatinib should not interfere with ligand-induced ErbB4/ErbB4 homotypic signaling or through ErbB4/ErbB3 heterotypic signaling. This may explain why lapatinib has emerged as a drug, with little or no cardiac toxicity [50].

Afatinib and neratinib are two second-generation irreversible tyrosine kinase inhibitors. Afatinib is highly selective for EGFR/ErbB1 and ErbB2, whereas neratinib also inhibits ErbB4, carrying the risk of block both homotypic and heterotypic signaling (Fig. 6c). No significant cardiac dysfunction in phase I and II trials have been reported for afatinib and neratinib. These early cardiac safety data are promising [50].

### Trastuzumab-emtansine

Trastuzumab-emtansine (also known as T-DM1) is an antibody conjugate consisting of the monoclonal antibody trastuzumab linked to cyctotoxic agent emtansine (Fig. 6d). Trastuzumab targets ErbB2-positive tumors, whereas emtansine is a highly potent antimicrotubule drug [29]. This toxic effect is restricted to ErbB2-expressing cells that result in very little neuropathy and no hair loss [11]. T-DM1 has a better overall safety profile compared with trastuzumab. No significant cardiotoxicity was observed with T-DM1 in patients, previously treated with trastuzumab and a taxane [24]. Obviously, T-DM1 has never been tested in treatment schedules that co-administrated anthracyclins and T-DM1.

### MM-111

MM-111 is a new bispecific antibody and polypeptide fusion protein of two human scFv antibodies linked to modified human serum albumin (Fig. 6e). The resulting molecule, MM-111, forms a trimeric complex with ErbB2 and ErbB3, inhibiting ErbB3 signaling and showing antitumor activity in preclinical models that are dependent on ErbB2 overexpression [38]. By its design, MM-111 blocks the activity of ligand-dependent and ligand-independent ErbB2/ErbB3 complexes in tumor cells, hence, recapitulating the effect of combining trastuzumab and pertuzumab or lapatanib, albeit in a completely different manner. A recent study demonstrated that MM-111 inhibition of ligand-activated ErbB3 phosphorylation is superior to pertuzumab, and that the combination of MM-111 with trastuzumab more effectively inhibits tumor cell growth than pertuzumab plus trastuzumab [38]. The underlying reason is that pertuzumab merely indirectly inhibits ErbB3 activation by precluding ErbB2 dimerization. In tumor cells with a high number of ErbB2, inhibition of ErbB2 by pertuzumab may be incomplete. By contrast, MM-111 inactivates ErbB3 in a direct way.

A major advantage of MM-111 is its specific binding to ErbB2/ErbB3 complexes. Since these complexes are absent or at least of minor abundance and/or physiological importance in the heart, it is very unlikely that MM-111 interferes with the physiological function of NRG-1 in the heart. Accordingly, at least from this perspective, the cardiotoxic profile of MM-111 should be safe.

## Conclusions

The crucial position of ErbB2 at the cross road between cancer and heart failure has made it an attractive therapeutic target in both oncology and cardiology. Growing knowledge of ErbB2 signaling as well as smart drug design has gradually led to the development of therapeutics to target ErbB2 signaling in the right cell type, for safe use in cancer and heart failure. Molecules, such as bivalent NRG-1 or MM-111, are exemplary for how a combination of a detailed understanding of biology and smart drug design can lead to fascinating progress in a complex medical field. Further clinical introduction of these drugs is ongoing and will tell whether these drugs can live up to the expectations.
